# Interplay between Janus Kinase/Signal Transducer and Activator of Transcription Signaling Activated by Type I Interferons and Viral Antagonism

**DOI:** 10.3389/fimmu.2017.01758

**Published:** 2017-12-11

**Authors:** Yuchen Nan, Chunyan Wu, Yan-Jin Zhang

**Affiliations:** ^1^Department of Preventive Veterinary Medicine, College of Veterinary Medicine, Northwest A&F University, Yangling, China; ^2^Molecular Virology Laboratory, VA-MD Regional College of Veterinary Medicine, Maryland Pathogen Research Institute, University of Maryland, College Park, MD, United States

**Keywords:** interferons, Janus kinase/signal transducer and activator of transcription signaling, Janus kinases, signal transducer and activator of transcriptions, viral antagonism, viral attenuation

## Abstract

Interferons (IFNs), which were discovered a half century ago, are a group of secreted proteins that play key roles in innate immunity against viral infection. The major signaling pathway activated by IFNs is the Janus kinase/signal transducer and activator of transcription (JAK/STAT) pathway, which leads to the expression of IFN-stimulated genes (ISGs), including many antiviral effectors. Viruses have evolved various strategies with which to antagonize the JAK/STAT pathway to influence viral virulence and pathogenesis. In recent years, notable progress has been made to better understand the JAK/STAT pathway activated by IFNs and antagonized by viruses. In this review, recent progress in research of the JAK/STAT pathway activated by type I IFNs, non-canonical STAT activation, viral antagonism of the JAK/STAT pathway, removing of the JAK/STAT antagonist from viral genome for attenuation, and the potential pathogenesis roles of tyrosine phosphorylation-independent non-canonical STATs activation during virus infection are discussed in detail. We expect that this review will provide new insight into the understanding the complexity of the interplay between JAK/STAT signaling and viral antagonism.

## Introduction

Interferons (IFNs) are a group of secreted proteins that play key roles in host antiviral immunity. IFNs are typically induced by the activation of host pattern-recognition receptors (PRRs), mainly RIG-I-like receptors (RLR) and toll-like receptors (TLR), during viral infection (1, 2). To date, three types of IFNs (I, II, and III) have been identified. Type I IFNs (referred to as IFNs in this review) compose the largest IFN family (3). Type II IFNs comprise only IFN-γ, which is unrelated to type I IFNs because it uses different receptors and is encoded by a different chromosomal locus (3, 4). Type III IFNs were recently discovered and comprise IFN-λ1, IFN-λ2, IFN-λ3, and IFN-λ4 (4, 5). IFN-λ signals through a unique receptor but activates the same pathway as that of type I IFNs (4, 6, 7). The classification of different IFN types along with their corresponding receptors is summarized in Table 1.

**Table 1:** Classifications of interferons (IFNs) and their receptors.

Type	Subtype	Receptor
Type I	IFN-α (13 subtypes)	IFNAR1 and IFNAR2
	IFN-β, IFN-ε, IFN-κ, and IFN-ω	
	IFN-δ (swine), IFN-τ (ruminant), and IFN-ζ (mice)	
Type II	IFN-γ	IFNGR1 and IFNGR2
Type III	IFN-λ1, IFN-λ2, IFN-λ3, and IFN-λ4	IFNLR1 and IL-10R2

All types of IFNs are capable to activate the Janus kinase/signal transducer and activator of transcription (JAK/STAT) pathway. In this review, recent progress of canonical or non-canonical activation JAK/STAT pathway, viral antagonism of the JAK/STAT pathway, removing of the JAK/STAT antagonist from viral genome for virus attenuation, and the potential pathogenesis roles of tyrosine phosphorylation-independent non-canonical STATs activation during virus infection are discussed in detail to provide new insight to understand the interplay between JAK/STAT signaling and viral antagonism.

## IFN-Activated Canonical JAK/STAT Pathway

Like other cytokines, IFNs bind their receptors and lead to the activation of certain signaling pathways, mainly the JAK/STAT pathway (8). Based on analysis of the flanking 5′-regions of genes locus in response to IFN, an IFN-stimulated response element (ISRE) was identified (9, 10). Probing the lysate of IFN-treated cells using ISREs, a cellular factor (named ISGF3) consisting of four preexisting proteins with sizes of 48, 84, 91, and 113 kDa was identified (10). Three of the four proteins with sizes of 91, 84, and 113 kDa were thought to belong to the same protein family and are currently known as STAT1α, STAT1β, and STAT2, respectively. The 48-kDa protein was later renamed interferon regulatory factor 9 (IRF9) (11). The size differences of STAT1α and STAT1β are due to alternative splicing of the same gene product (12). Moreover, the involvement of a kinase in IFN-α-induced IFN-stimulated gene (ISG) expression led to the discovery of the JAK family and STAT phosphorylation (13).

There are four members of the JAK family: JAK1, JAK2, JAK3, and tyrosine kinase 2 (TYK2). They are all characterized as having a C-terminal catalytic domain and a related, but enzymatically inactive, pseudo-kinase or kinase-like domain (14). They also share sequence similarity in five additional domains in the N-terminal region (15). The seven domains are now called Janus homology domains (JHD) 7 to 1 from the N- to C-terminal region of the JAKs (15). The four JHDs in N-terminal regions of JAKs (JHD7 to JHD4) are also called band 4.1 domains (C-terminus of JAKs) due to their homology to the band 4.1/ezrin–radixin–moesin protein family (16). The band 4.1 domains and Src homology 2 (SH2) domain (JHD5) are responsible for receptor binding (17). The pseudo-kinase domain (JHD2) is thought to regulate the kinase activity of JAKs *via* an interaction with the kinase domain (JHD1) (18).

**Figure 1:**
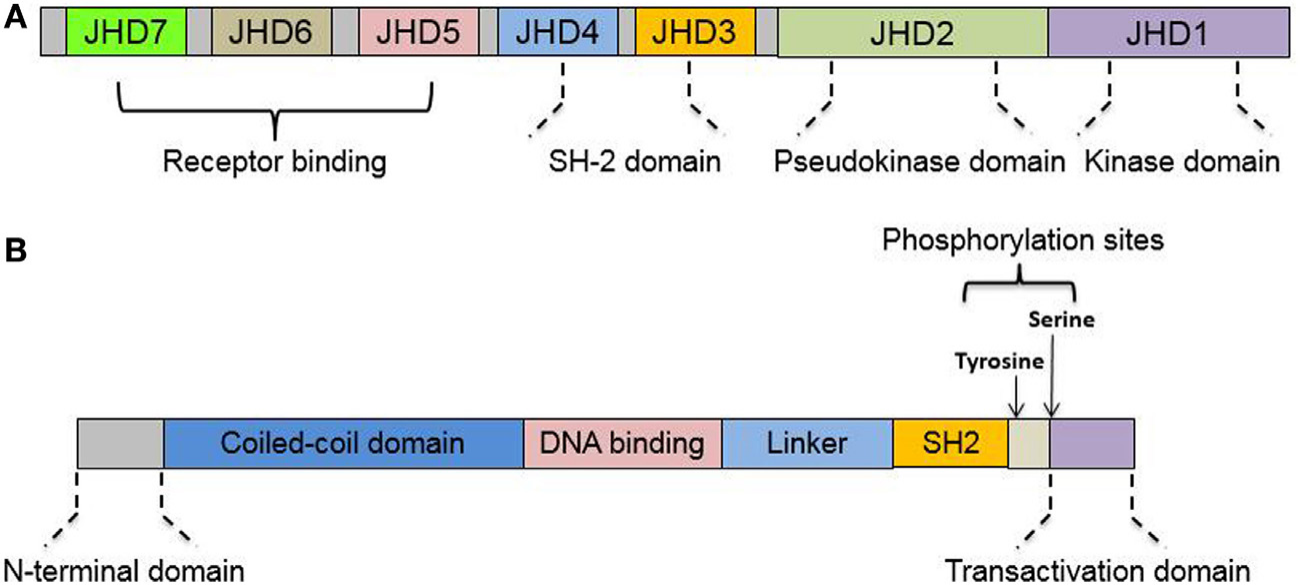
Protein domains of Janus kinase (JAK) and signal transducer and activator of transcription (STAT). **(A)** Structure illustration of structural and functional domains in JAK. JAKs share seven regions of high homology [Janus homology domains (JHD) 1–7], JHD1 has been shown to encode the kinase while JHD2 represents a pseudo-kinase domain to regulate JH1 catalytic activity. **(B)** Structure illustration of structural and functional domains in STAT. All STATs share six conserved domains, including an N-terminal domain, a coiled-coil domain, the DNA-binding domain, a linker domain, an Src homology 2 (SH2) domain, and C-terminal transactivation domain. See text for details.

The STAT family in mammalian hosts has seven members: STAT1, 2, 3, 4, 5A, 5B, and 6 (19). Sequence analysis shows remarkable similarity among the STAT genes, with the exception of STAT2 (19). All STATs share a very similar structure: an N-terminal domain, a coiled-coil domain, a DNA-binding domain (DBD), a linker domain, an SH2 domain, and a transactivation domain (TAD) (20). Schematic illustration of JAKs and STAT protein structure is listed as Figure 1. However, isoforms resulting from similar patterns of RNA splicing or protein proteolytic processing were reported for all STATs except STAT2 (21–24). These STAT isoforms lack the C-terminal TAD domain, which implies a regulatory role for STAT activation (22, 25, 26).

Janus kinases are generally non-covalently associated with the cytoplasmic tail of specific receptors. Upon cytokine binding, receptor dimerization or oligomerization leads to JAK apposition and autophosphorylation on tyrosine residues, releasing their intrinsic catalytic activity. Tyrosine phosphorylation of cytokine-receptor cytoplasmic domains by activated JAKs then provides binding sites for the SH2 domains of the STAT proteins. The STATs are then recruited to the JAKs, whereupon they are phosphorylated at a tyrosine residue (near residue 700 of their 750–850 aa-long sequence) (15). Upon activation, STAT/STAT interactions occur immediately through reciprocal SH2 interactions (27). All STATs are able to form homodimers, and the formation of STAT heterodimers is different depending on cytokines activating upstream signaling (15, 28).

STAT1 and STAT2 are the major players in type I IFN-mediated signaling (28). Other complexes induced by type I IFNs include STAT1–STAT1, STAT3–STAT3, STAT4–STAT4, STAT5–STAT5, and STAT6–STAT6 homodimers as well as STAT1–STAT3, STAT1–STAT4, STAT1–STAT5, STAT2–STAT3, and STAT5–STAT6 heterodimers (28). Karyopherin α1 (KPNA1) is the essential importin for the nuclear transport of phosphorylated STAT1 (29). STAT1 possesses a non-classical NLS, and KPNA1 binds between two STAT1 monomers, with two major binding determinants in the SH2 and DBDs (30). In addition to mediating IFN signaling, STATs are also responsible for transducing signals for several families of cytokines (Table 2).

**Table 2:** Signal transducer and activator of transcription (STATs) for different cytokine signaling.

Type	Cytokines
STAT1	Type I, type II, and type III interferons (IFNs)
STAT2	Type I, type II, and type III IFNs
STAT3	IL-6 (IL-6, IL-11, IL-31, LIF, CNTF, CLC/CLF, NP, CT1, and OSM) and IL-10 (IL-10, IL-19, IL-20, IL-22, IL-24, and IL-26) families, G-CSF, leptin, IL-21, and IL-27
STAT4	IL-12
STAT5A and STAT5B	IL-3 family (IL-3, IL-5, and GM-CSF), IL-2 family (IL-2, IL-7, TSLP, IL-9, IL-15, and IL-21), growth hormone, Epo (erythropoietin), and Tpo (thrombopoietin)
STAT6	IL-4 and IL-13

## Tyrosine Phosphorylation-Independent Non-Canonical STAT Activation

The phosphorylation of tyrosine residues (near residue 700) in STATs is generally considered an essential step in the canonical activation of the JAK/STAT pathway induced by IFN or other cytokines (31). However, STATs can also be phosphorylated on serine residues in the C-terminal TAD (31). Initially, phosphorylation of serine residue in the TAD domain was considered to contribute to the maximal transcriptional activity of STAT in addition to tyrosine-dependent STAT activation (32). However, in recent years, STATs without tyrosine phosphorylation have been found to undergo continuous nuclear import/export and to contribute to alternative gene expression as non-canonical STAT activation (33, 34). It was shown that EBV the can specifically promote the expression of several ISGs including STAT1 without stimulating IFN induction or JAK/STAT activation but depends its early lytic nuclear protein SM protein (35). This observation challenges the canonical model of STATs activation, which generally views expression of ISGs as a consequence of STATs activation requires tyrosine phosphorylation.

Moreover, it has been demonstrated that unphosphorylated STAT1 and STAT2 with IRF9 can form unphosphorylated ISGF3 (U-ISGF3) (36). U-ISGF3 formation requires high levels of IRF9, STAT1, and STAT2 without tyrosine phosphorylation, and U-ISGF3 could also be induced by low level IFN-β. It was proposed that phosphorylated ISGF3 drives the first rapid-response phase, while U-ISGF3 drives the second prolonged response by binding to distinct ISREs, which are different from the ISREs in the rapid phase (36). Moreover, recent reports also demonstrated that U-ISGF3 drives the constitutive expression of ISGs to protect against viral infection under homeostatic conditions (37, 38). In addition to U-STAT1 and U-ISGF3, tyrosine unphosphorylated form of STAT as transcription activator has been reported for other STATs as well and is proposed to play roles in cytokine signaling, cell proliferation, hematopoietic differentiation, and cancer prognosis (34, 39–43). Moreover, it appears that unphosphorylated STATs other than STAT1/2 can form homodimers or heterodimers (41).

In addition to unphosphorylated STATs, mono-phosphorylation of the serine residues of different STATs has been reported (as serine phosphorylation of the TAD without tyrosine phosphorylation) in recent years (31, 44), which represents a novel non-canonical pathway of STAT activation (45). Unlike JAKs inducing tyrosine phosphorylation, the kinase involved in the serine phosphorylation of STATs is still unclear and might be involve the p38 MAPK pathway or occur *via* ERK or cyclin-dependent kinase 8 (CDK8) (31, 46–48). Serine mono-phosphorylation of STAT1 has been investigated more often than that of other STATs (49). In a mouse model of bacterial infection, a modest gain-of-function in antibacterial immunity was found in a STAT1Y701F mutant compared with that in Stat1^−/−^ mice (33, 49), suggested that serine mono-phosphorylated STAT1 at S727 site might contribute the partial restoration of antibacterial immunity in STAT1Y701F mutated mice. Notably, a STAT1Y701F mutant partially retained NK cell cytotoxicity, in contrast to a complete loss in Stat1^−/−^ mice. However, the NK maturation defect in the STAT1Y701F mutant was similar to that found in Stat1^−/−^ mice. A single mutation of serine phosphorylation (STAT1-S727A) enhances NK cell cytotoxicity against a range of tumor cells (50, 51). In acute myeloid leukemia (AML), it appears that serine mono-phosphorylation and nuclear translocation of STAT1 were promoted by ERK, with certain chemokines and ISGs upregulated by STAT1-S727 (48). Moreover, a higher level of serine mono-phosphorylation of STAT5 was found in AML and appears to be CDK8 dependent (52).

The involvement of STAT2 in non-canonical STAT activation is also interesting. Phosphorylation of STAT2 at serine 734 appears to negatively regulate the IFN-α-induced antiviral response (53). However, when IFN stimulation is lacking, STAT2 (unphosphorylated form) constitutively binds to activated STAT1, thus specifically precluding the nuclear translocation of STAT1 in response to IFN-γ, IL-6, and IL-27 (54). Moreover, STAT2 can form an ISGF3-like complex with IRF9 in the absence of STAT1 to evoke a prolonged ISGF3-like transcriptional response and antiviral activity (55).

The current understanding of tyrosine phosphorylation-independent non-canonical STAT activation is still limited, and more investigation is needed. The existing literature presents investigations of U-STAT that mainly focus on tyrosine phosphorylation and rarely on serine phosphorylation (38, 56, 57). However, it is not known whether the function of the serine mono-phosphorylation of STATs correlates with U-STATs or the transcription complex such as U-ISGF3 (44). It is also unclear whether U-STATs and serine mono-phosphorylated STATs are functionally equal or whether they actually have unique functions. Therefore, the functions of U-STATs and serine mono-phosphorylated STATs should be delineated in future studies. Moreover, except for serine phosphorylation in the TAD domain of STATs, more phosphorylation sites in STATs have been reported, such as serine 287, threonine (T) 800, and T597 in STAT2 (58–60). The functions of these novel phosphorylation sites and their correlation with non-canonical STAT activation will be further explored in the next decade.

## Regulation of the JAK/STAT Pathway

### Protein Regulators of the JAK/STAT Pathway

As they are essential mediators of cytokine or hormone signaling, the activation of STATs is tightly regulated. The suppressor of cytokine signaling (SOCS) family comprises well-defined regulator of the JAK/STAT pathway (61), including SOCS1 to SOCS7 and CIS (cytokine-induced SH2 containing protein) (62). All SOCS proteins share a common structure with an SH2 domain and a C-terminal SOCS box domain (62). The SOCS box domain is critical for the proteasome-mediated degradation of SOCS-associated proteins (62). Meanwhile, SOCS1 and SOCS3 contain an additional kinase inhibitory region for the inhibition of kinase activity (63). Therefore, SOCS members inhibit JAK/STAT *via* various routes, such as blocking STAT recruitment to the cytokine receptor, targeting STATs for proteasome degradation, binding to JAKs, and targeting JAKs for proteasome degradation (64–66).

In addition to the well-defined SOCS family, JAK/STAT signaling can also be regulated by cysteine-based protein tyrosine phosphatases (PTPs), such as by the dephosphorylation of pTyr residues in the JAK/TYK activation loop or phosphorylation sites in the cytoplasmic domains of the cytokine receptors (67). However, the specificity and detailed mechanism of the PTP-mediated regulation of JAK/STAT still require further investigation. The protein inhibitor of activated STATs (PIASs) is another class of JAK/STAT regulators but is proposed to have more a complicated function due to their function as SUMO E3 ligases (68–70). The SUMOylation of STATs by PIAS has also been identified as a modulatory mechanism (71, 72). It was demonstrated that the SUMOylation of STAT1 obstructs the phosphorylation of a proximal tyrosine residue, which leads to semi-phosphorylated STAT dimers, which competes with their fully phosphorylated counterparts and interferes with the JAK/STAT pathway (72).

### Posttranslational Modification (PTM) of STATs

In addition to the SUMOylation of STATs by PIAS, other PTMs have been suggested to regulate STAT activation both positively and negatively (73). The acetylation of STAT1, STAT2, STAT3, STAT5b, and STAT6 has been identified and reviewed elsewhere (74). The acetylation of STATs is dependent on the balance between histone deacetylases (HDACs) and histone acetyltransferases, such as CBP/p300 (74). Generally, the acetylation of STATs increases the DNA-binding affinity and promotes transcription activation and STAT dimerization, as acetylation of STATs can occur at various lysine residues located in different domains (74, 75). Moreover, it is interesting that SUMOylation and acetylation can occur on the same lysine residue in STAT5 (lysine 696) and are mutually exclusive with each other (76, 77), which suggests that SUMOylation and acetylation might maintain a balance in STAT function.

The arginine- and lysine-based methylation of STATs is another method of regulating STAT activation (78), but it is complicated by both negative and positive roles for STAT activation. Arg-31 methylation was shown to be required for STAT1 transcriptional activation (79). However, a later study reported that the inhibition of STAT1 arginine methylation at Arg-31 results in a prolonged half-life of STAT1 tyrosine phosphorylation (80), which suggests that Arg-31 methylation negatively regulates STAT1 activation. Moreover, methylation at Arg-27 of STAT6 is necessary for optimal STAT6 phosphorylation, nuclear translocation, and DNA-binding activity (81). Recently, a new methylation site in STAT1 (Lys-525) was identified that is required for STAT1-mediated antiviral immunity (82). Moreover, STAT3 is reversibly methylated on Lys-140 and Lys-180 by the histone methyl transferases SET9 and EZH2, respectively (83, 84). Mass spectroscopy analysis shows that unphosphorylated STAT3 (U-STAT3) is acetylated on Lys-685, and the integrity of Lys-685 is required for the expression of most U-STAT3-dependent genes (85).

In addition to methylation and acetylation, ISGylation—the conjugation of targets by interferon stimulated gene 15 (ISG15, an ubiquitin-like protein)—has been shown to positively regulate IFN signaling (86, 87). An earlier study revealed that mice lacking UBP43, a protease that removes ISG15 from conjugated targets, are hypersensitive to type I IFN (88). A recent study suggested that ISGylation of STAT1 increases the stability of STAT1 and prevents the premature termination of the immune response in LPS-stimulated microglia (89).

The PTM of STATs still requires additional investigation because cross talk between methylation, SUMOylation, and acetylation remains unclear. Moreover, a recent study demonstrated that the inhibition of HDAC enhances STAT acetylation but blocks NF-κB signaling during renal inflammation and fibrosis in haplotype Npr1^+/−^ male mice (90). Therefore, cross talk between the JAK/STAT pathway and the NF-κB pathway under the same PTM conditions is complicated and requires further exploration. Although dysregulation of PTMs in STATs during viral infection has been reported, and modulation of STATs PTM may be employed by virus to evade from antiviral responses mediated by IFNs (91, 92), there has been little investigation regarding whether virus infection can affect the PTMs of STATs to regulate the JAK/STAT pathway.

### Regulation of the JAK/STAT Pathway *via* Host MicroRNAs (miRNA)

Host miRNAs are small non-coding RNAs ~22 nucleotides in length that control gene expression by binding to the 3′-untranslated region of a target mRNA, thereby affecting mRNA stability and/or translation (93, 94). The regulation of the JAK/STAT cascade by miRNA emerges as a novel mechanism for the development and progression of many diseases (95). The miRNAs with a confirmed target in the JAK/STAT pathway are summarized in Table 3. In addition to miRNAs with well-defined targets, certain miRNAs with unidentified targets contribute to enhanced or attenuated IFN signaling, such as miRNA-26a, miRNA-146a, and miRNA-9 (96–99). A recent report showed that miRNA-551b-3p binds to the STAT3 promoter and promotes STAT3 expression, which enhances STAT3-mediated signaling without directly targeting mRNA encoding proteins in STAT3 signaling (100), implying a novel mechanism for regulating the JAK/STAT pathway *via* miRNA.

**Table 3:** List of microRNA (miRNA) regulating Janus kinase/signal transducer and activator of transcription signaling (JAK/STAT) pathway with confirmed targets.

Targets	miRNA no.	Reference
Interferons receptors	miRNA-29a; miRNA-208b; and miRNA-499a-5p	(101, 102)
JAK1	miRNA-30c and miRNA-373	(103, 104)
JAK2	miRNA-216a and miRNA-101	(105, 106)
STAT1	miRNA-450a-5p, miRNA-28-5p, miRNA-145, miR-146a, miR-150, and miR-223	(107–110)
STAT2	miR-221/222	(111)
STAT3	miR-124	(112)
STAT4	miR-132, miR-212, and miR-200a	(113).
STAT5b	miR-150	(114)
SOCS2	miRNA-424-5p	(115)
SOCS3	miRNA-122	(116)

Moreover, since most miRNAs as JAK/STAT regulators were discovered in cancer cells or other disease conditions (i.e., virus infection, apoptosis, and inflammation), it remains unknown if these miRNAs represent a universal role for regulating the JAK/STAT pathway under normal physiological conditions or act only under the diseased states. Meanwhile, other non-coding RNA molecules, such as long non-coding RNA and circular RNA, have been confirmed to play a role in the regulation of the innate immune response (117–120). Their roles in regulation of the JAK/STAT pathway remain unknown and require further investigation.

## Viral Interference of IFN-Activated JAK/STAT Signaling

### Blocking the Binding of IFNs to Their Receptors or Targeting IFNs Receptors

**Figure 2:**
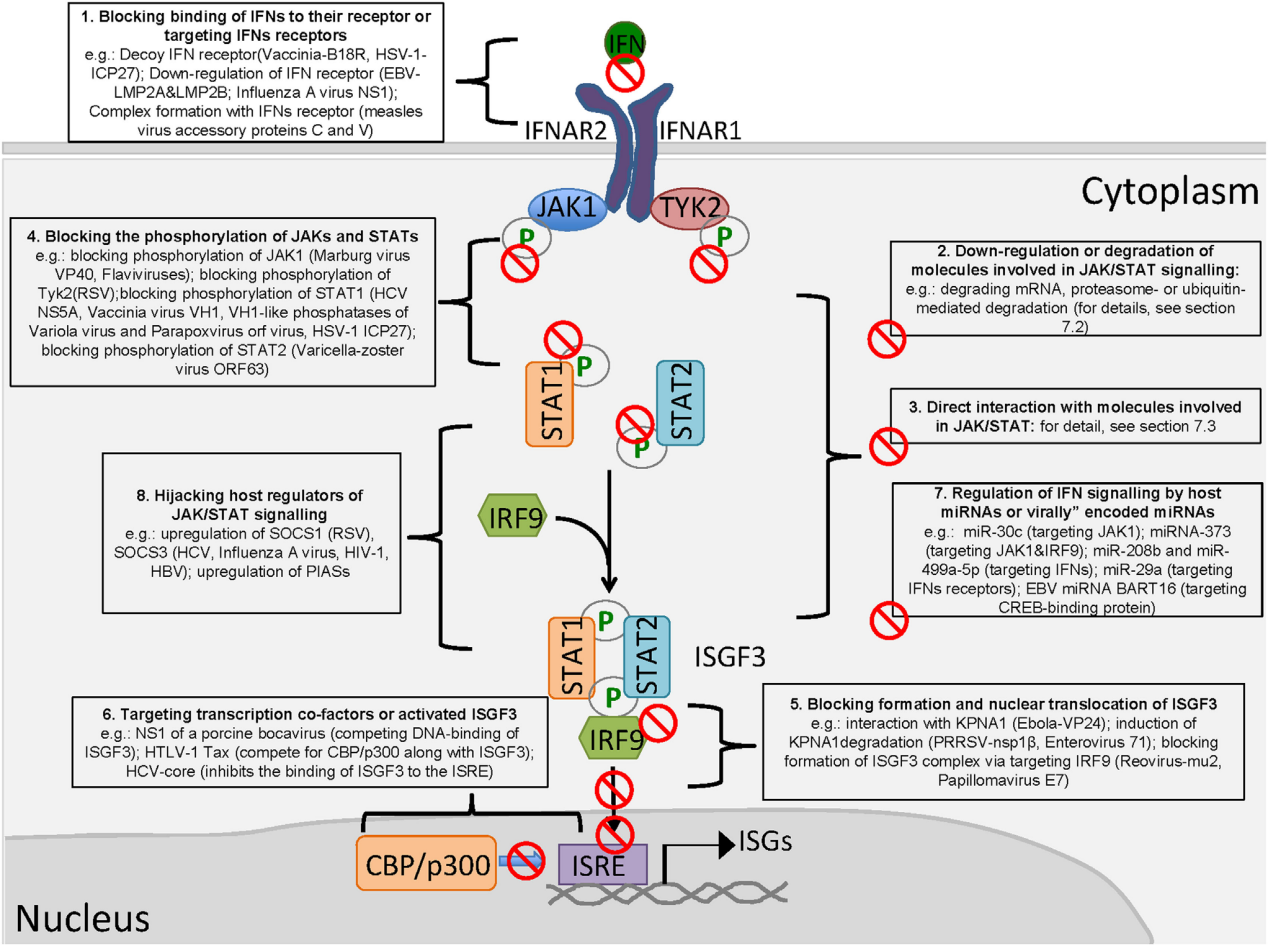
Viral antagonism of type I interferon (IFN)-activated Janus kinase/signal transducer and activator of transcription signaling (JAK/STAT) signaling. (1) Blocking binding of IFNs to the receptor; (2) downregulation or degradation of molecules involved in JAK/STAT signaling; (3) direct interaction with molecules involved in JAK/STAT signaling; (4) blocking the phosphorylation of JAKs and STATs; (5) blocking the formation and nuclear translocation of ISGF3; (6) targeting transcription cofactors or activated ISGF3; (7) regulation of IFN signaling by host microRNAs (miRNAs) or virally encoded miRNAs; and (8) hijacking host regulators of JAK/STAT signaling.

Viruses employ various strategies to antagonize the JAK/STAT pathway and facilitate their own replication (Figure 2). The binding of IFNs to their receptors is the first step in the activation of JAK/STAT signaling. Vaccinia virus encodes the secreted protein B18R that possesses a region with three immunoglobulin domains with high levels of homology to IFNAR1, and B18R is able to serve as a soluble receptor to prevent an IFN-mediated antiviral effect (121, 122). Similar proteins (ICP27) were identified from HSV-1 (123). Although no RNA virus-encoded decoy receptor has been identified, measles virus accessory proteins C and V can form a complex with IFNAR1 to block the activation of JAK1 (124). Similarly, the regulator of IFN function protein of Kaposi’s sarcoma-associated herpesvirus (KSHV) blocks the IFN response by directly interacting with IFNAR (125). Moreover, the latent membrane proteins LMP2A and LMP2B of the Epstein–Barr virus modulate IFN signaling by accelerating IFNAR turnover (126). Meanwhile, influenza A virus NS1 reduces IFNAR expression at the transcriptional level (127).

### Downregulation or Degradation of Molecules Involved in JAK/STAT Signaling

The downregulation of molecules responsible for IFN-activated signal transduction is a common mechanism employed by viruses. HCMV can decrease the JAK1 level (128). HSV-1 appears to degrade cellular mRNAs, leading to a partial reduction of JAK1 and STAT2 (129). E1A of adenovirus causes a reduction of STAT1 and IRF9 (130). HCV degrades STAT1 and STAT3 (131, 132). Furthermore, porcine epidemic diarrhea virus targets STAT1 for the proteasome-dependent degradation (133).

It appears that most members of the *Rubulavirus* genus of the subfamily *Paramyxovirinae* have acquired the ability to degrade STAT1 or STAT2 by their accessory V protein, which is encoded by the P gene (134). The V protein of human parainfluenza virus 2 causes the degradation of STAT1 and STAT2 (135–138). The DENV NS5 protein can also mediate STAT2 degradation *via* the ubiquitin-proteasome pathway (139), consistent with a similar report regarding NS5 of the Zika virus (ZIKV), another flavivirus (140, 141). The RSV NS1 protein induces the degradation of STAT2 *via* an elongin-cullin E3 ligase (142). The 3C-like protease encoded by porcine deltacoronavirus nsp5 cleaves STAT2 at the Q685 and Q758 sites (143).

As a component of ISGF3, IRF9 is also targeted by viruses. Rotavirus NSP1 mediates the degradation of IRF9 (144). In addition to blocking STAT2 phosphorylation, the varicella-zoster virus (VZV) ORF63 product induces the degradation of IRF9 (145). HCMV reduces the levels of JAK1 and IRF9 in human embryonic lung fibroblasts (146).

### Direct Interaction with JAK/STAT Signaling Molecules

Some viruses encode proteins to interact with both JAKs and STATs to inhibit the phosphorylation of STATs. The E6 protein of HPV18 (human papilloma virus) interacts with the JH6–JH7 domains of Tyk2, which are critical for Tyk2 and IFNAR1 interaction, to prevent Tyk2 phosphorylation (147). The V protein of paramyxovirus and the measles virus binds JAK1 to inhibit downstream signaling (148, 149). The accessory factors (V, C, P, etc.) expressed by the P gene of paramyxoviruses disrupt STAT signaling *via* various mechanisms, including direct interaction with STATs (134). The C protein of the Sendai virus inhibits IFN signaling *via* binding STAT1 to block the formation of a heterodimer or homodimer (150). Meanwhile, C protein is able to induce its mono-ubiquitination and degradation of STAT1 (151). Non-structural proteins of severe fever with the thrombocytopenia syndrome virus interact with STAT2 and sequester STAT1/STAT2 and STAT1 into viral inclusion bodies to impair IFN signaling (152). Moreover, VP24 of the Ebola virus can form a complex with STAT1 *via* a novel, pyramidal fold structure, as revealed by structure analysis (153).

### Blocking the Phosphorylation of the JAKs and STATs

Since the phosphorylation of STAT1 and STAT2 by JAK1 and Tyk2 is the key step for their activation, both JAKs and STATs are frequently targeted by virally encoded antagonists to inhibit their activation. RSV impairs IFN-β-mediated STAT1 signaling through the inhibition of TYK2 phosphorylation (154). Similarly, VP40 of the Marburg virus antagonizes JAK1 and STAT1 phosphorylation (155). All flaviviruses examined to date, including the West Nile virus (WNV), Japanese encephalitis virus, Langat virus, and Dengue virus, can suppress JAK/STAT signaling by inhibiting JAK phosphorylation (156–159). This suppression blocks the downstream phosphorylation of STAT1 and STAT2. In addition, some viruses directly target STATs to inhibit phosphorylation. HCV NS5A disrupts STAT1 phosphorylation and suppresses IFN signaling (160, 161). Rotavirus NSP1 inhibits IFN-mediated STAT1 phosphorylation (162).

Viruses also encode specific phosphatases to dephosphorylate STAT1 at tyrosine 701 to inhibit IFN signaling. Vaccinia virus VH1 blocks both IFN-α- and IFN-γ-stimulated signaling (163), and the dimerization of VH1 is essential for its phosphatase activity on STAT1 (164). Moreover, VH1-like phosphatases have been identified from other DNA viruses, such as the highly virulent variola virus (Smallpox) and parapoxvirus orf virus, which belong to the poxvirus and baculovirus families, respectively (165, 166). VZV blocks STAT2 phosphorylation *via* its ORF63 product (145). The HSV-1 immediate-early gene ICP27 downregulates STAT1 phosphorylation by retaining STAT1 in the nucleus *via* an unknown mechanism (167).

### Blocking the Formation and Nuclear Translocation of ISGF3

Nuclear translocation of the ISGF3 complex is another antagonizing target for viruses. The multifunctional P protein of the rabies virus inhibits STAT1 nuclear translocation by directly interacting with STAT1 but without affecting STAT1 phosphorylation (168). As KPNA1 is the essential importin for the nuclear transport of phosphorylated STAT1 (29), it has been frequently targeted. VP24 of the Ebola virus is known to bind to KPNA1 to disrupt the interaction between phosphorylated STAT1 and KPNA1, thereby preventing STAT1 nuclear translocation (169). VP24 interacts with KPNA1 but not KPNA2, KPNA3, or KPNA4 (169). However, a recent report suggested that VP24 binds KPNA5 to antagonize IFN signaling (170). Moreover, another study suggested that VP24-karyopherin-α binding affinities differ among different Ebola virus species (171), which may contribute to the differences in virulence.

The porcine reproductive and respiratory syndrome virus (PRRSV) nsp1β protein is another KPNA1 antagonist that inhibits IFN signaling (172, 173). However, no direct interaction between KPNA1 and nsp1β of PRRSV has been detected (173). Instead, nsp1β is able to induce the ubiquitin-mediated degradation of KPNA1, thus leading to the blockage of ISGF3 nuclear transportation (173). The 3Cpro of FMDV contributes to the degradation of KPNA1 in ubiquitination depended manner as that of nsp1β of PRRSV to block STAT1/STAT2 nuclear translocation (174). Moreover, a recent report demonstrated that enterovirus 71 suppresses IFN responses by inducing KPNA1 degradation in a manner similar to that of PRRSV (175).

In addition, papillomavirus E7 oncoprotein binds to IRF9 to block the formation of the ISGF3 complex (176). Moreover, the mu2 protein of reovirus blocks the nuclear accumulation of IRF9, a novel mechanism for the inhibition of IFN signaling (177).

### Targeting Transcription Cofactors or Activated ISGF3

The activation of ISG transcription by ISGF3 is the last step of IFN signaling and involves transcription cofactors or coactivators. In contrast to the upstream steps, virus-mediated inhibition of transcription activation of ISGF3 has been less investigated. NS1 of a porcine bocavirus inhibits the DNA-binding activity of ISGF3 by interacting with the DBD of IRF9 (178). The TAX protein of human T-cell leukemia virus type 1 competes with ISGF3 for the coactivator CBP/p300, thus inhibiting IFN signal transduction (179). Similarly, the tegument protein VP16 blocks the recruitment of the coactivator CBP to inhibit IFN induction and NF-κB activation (180). Moreover, HCV inhibits the binding of ISGF3 to the ISRE element *via* its core protein (181).

### Regulation of IFN Signaling by Hijacking Host miRNAs or Virally Encoded miRNAs

The regulation of proteins in the JAK/STAT cascade by miRNAs is a novel mechanism involved in the progression of many diseases (95, 182, 183). For example, miR-30c upregulated during PRRSV infection dampens signaling by IFNs by targeting JAK1 (104). For HCV, the upregulation of miRNA-373 suppresses JAK1 and IRF9 expression (103). HCV-induced miR-208b and miR-499a-5p also dampen type I IFN signaling in HCV-infected hepatocytes by directly downregulating the expression of IFNAR (101). RSV non-structural protein 1 induces miR-29a to downregulate IFNAR (102). Human T-cell lymphotropic virus type 1 (HTLV-1) downregulates miR-150 and miR-223 to promote the expression of STAT1, as constitutive activation of STAT1 is required for the continuous proliferation of HTLV-1-transformed cells (109). Meanwhile, certain miRNAs with unidentified targets contribute to enhanced IFN signaling. miR-26a is such a miRNA (96, 97). Influenza A virus downregulates miR-26a to block IFN signaling (184). miR-122 contributes to IFN signaling by inhibiting SOCS1 expression. HBV suppresses miR-122 to inhibit IFN signaling (116).

Recently, a novel viral-encoded miRNA was shown to regulate IFN signaling. Virally encoded miRNA targeting molecules involved in IFN induction pathways (RLR and TLR signaling) have been reported for years (185–187). However, EBV miRNA BART16 is the first virally encoded miRNA that has been shown to interfere with type I IFN signaling *via* targeting the CREB-binding protein, a key transcriptional coactivator in EBV-transformed B cells and gastric carcinoma cells (188). Because the expression of virally encoded miRNAs in herpesvirus or other large DNA viruses is common, additional JAK/STAT-antagonizing miRNAs from these viruses may be identified in the future.

### Hijacking Host Regulators of JAK/STAT Signaling

The activation of the JAK/STAT pathway is regulated by the SOCS protein family, which can be functionally “hijacked” by viruses to promote virus replication (189). The core protein of HCV induces SOCS3 expression when overexpressed in HepG2 cells (190). A further study confirmed that SOCS3 expression is increased in HCV-infected HepG2 cells and in the peripheral lymphocytes of HCV-infected individuals (191). Similarly, influenza A virus induces SOCS3 expression (192), and RSV upregulates SOCS1 (193). Furthermore, the Tat protein of HIV-1, a regulatory protein for viral transcription enhancement, contributes to the immune evasion of HIV by inducing SOCS3 expression (194). For DNA viruses, HSV-1 induces the upregulation of SOCS1 in keratinocytes (195). HBV X protein increases SOCS3 and protein phosphatase 2A (196).

### Viral Antagonism of Other STATs

In addition to STAT1 and STAT2, the other STATs activated by type I IFNs can be inhibited by virus infection, which has been less investigated; the role of other STATs in the IFN-mediated response still requires further investigation. Among the other STATs, STAT3 attracts more attention because both U-STAT3 and phosphorylated STAT3 are involved in the antiviral response activated by IFNs, and STAT3 specifically induces a subset of IFN-α-driven ISGs (197). OSM, a member of the IL-6 family, has also been shown to induce an antiviral response *via* activation of JAK/STAT3 signaling (34, 198). STAT3 is required for the optimal type I IFN response to HSV-1 in mice (199).

Viruses affecting JAK activation inhibit the phosphorylation of STAT3 (200). Influenza A virus NS1 and human metapneumovirus impede STAT3 phosphorylation in infected cells and simultaneously block type I IFN signaling (127, 201). In addition, tyrosine dephosphorylation of STAT3 at position Y705 in SARS coronavirus-infected Vero E6 cells was observed (202).

Some viruses directly target STAT3. The HCMV 72-kDa immediate-early 1 protein promotes the nuclear localization of STAT3 without robust phosphorylation, which disrupts the IL-6-induced expression of STAT3-dependent genes (203). The V protein of the mumps virus induces STAT3 ubiquitination and degradation to block IL-6 and v-Src signaling (204). Further investigation demonstrated that a single mutation of E95D in the V protein disengages its STAT3-targeting ability (205). The V protein of the measles virus also interferes with STAT3 activation *via* direct interaction (206). The P protein of the rabies virus binds activated STAT3 and inhibits its nuclear accumulation (207). PRRSV nsp5 induces the ubiquitin-mediated degradation of STAT3 to inhibit OSM-activated JAK/STAT3 signaling (198). HCV promotes STAT3 ubiquitination and degradation in a similar manner to PRRSV (132). Meanwhile, HCV increases SOCS3 expression, which is correlated with decreased STAT3 (208).

Less attention has been paid to the virally mediated antagonism of STAT4, 5, and 6. Currently, there is no virally encoded antagonist identified for these STATs. However, the available data suggest an important role for these STATs in the IFN-mediated response (209–211). STAT4 promotes IFN induction by blocking the CHIP-mediated ubiquitination and degradation of RIG-I (212). Moreover, the rs7574865 polymorphism of STAT4 (GG genotype) is significantly associated with a reduction in the sustained virologic response rate in patients receiving IFN therapy (213). However, the involvement of STAT4, 5, and 6 in IFN signaling still requires further investigation.

## JAK/STAT Antagonists and Virus Virulence: Implications for Virus Attenuation

Since the discovery of virally encoded IFN antagonists, it has been proposed that JAK/STAT pathway antagonism is a virulence factor that might offer a novel route of virus attenuation during vaccine development using a modified live virus. Mice lacking intact JAK/STAT signaling, such as IFN-receptor or STAT1 knockout mice, are more susceptible to virus infection than wild-type mice (214–217). In addition, *in vivo* data suggest that a fast type I IFN response protects astrocytes from flavivirus (tick-borne encephalitis virus, JEV, WNV, and ZIKV) infection (218). Notably, as reverse genetics technology facilitates the manipulation of virus genomes, point mutations or deletions of JAK/STAT antagonists have been explored to reduce viral virulence.

As described earlier, the measles virus P gene (encodes three proteins P, V, and C) is the major antagonist that interferes with IFN-mediated JAK/STAT signaling (219). Tyrosine 110, valine 112, and histidine 115 in the shared domain of the P and V proteins determine the STAT1-antagonizing function of these two proteins (219). A recombinant measles virus with a mutation at tyrosine 110 of the P protein fails to antagonize STAT1. Compared with the wild-type measles virus, the mutant virus leads to short-lived viremia, without a skin rash and other clinical signs in rhesus monkeys, which suggests attenuation.

Similar to these observations from the measles virus, another neurotropic virus, Sindbis virus, is capable of suppressing both type I and type II IFN-mediated responses by disrupting JAK/STAT signaling (220). However, two avirulent strains that are unable to cause detectable disease in adult mice were shown to be relatively inefficient inhibitors of STAT1/2 activation (220). Further analysis demonstrated that a single amino acid determinant, the Thr at aa 538 of nsP1 of Sindbis virus, restores the STAT1 inhibition of nsP1 when it is introduced into avirulent strains and is required for Sindbis virus virulence *in vivo* (220). Moreover, as another well-defined JAK/STAT antagonist, NS5 from the flavivirus member WNV has been linked to virulence. The NS5 protein from a naturally attenuated WNV strain was shown to be a poor suppressor of pY-STAT1. Restoration of a single residue in NS5 of attenuated WNV to the analogous residue in virulent WNV demonstrated efficient inhibition of STAT1 activation and conferred the virulence phenotype (221). Furthermore, as observed by crystallization, the STAT1 antagonist VP24 from a virulent Ebola Sudan strain has a novel, pyramidal fold structure, which contains a site on a particular face of the pyramid exhibits reduced solvent exchange when in complex with STAT1 (153). Compared with VP24 from the non-pathogenic Reston strain, this site is above two highly conserved pockets in VP24 that contain key residues previously implicated in Ebola virus virulence (153).

One of the most promising examples of virus attenuation promisingly based on removing the JAK/STAT antagonist is the influenza viruses. As described earlier, NS1 encoded by influenza A and B viruses antagonizes IFN-activated JAK/STAT signaling at multiple steps. Investigations into generating an attenuated influenza virus based on deleting NS1 from the influenza virus genome have been ongoing for decades. The complete deletion of NS1 from influenza A generates a viable virus, but the virus replicates at a much lower level (multiple log reduction of the viral titer) than wild type in normal MDCK cells; however, replication can be partially restored in IFN-deficient Vero cells (222). Meanwhile, the NS1-deleted influenza A virus maintains pathogenicity in STAT1 knockout mice but is no longer pathogenic in wild-type mice, suggesting that the attenuation of the influenza A virus by NS1 deletion is JAK/STAT dependent (222). Since then, more studies have been conducted. As a naturally truncated NS1 variant was identified and highly attenuated in the host (222, 223), to avoid over attenuation, partial deletions of NS1 (removing the C-terminal effector domain but maintaining the N-terminal RNA binding domain of NS1) were conducted to generate mutant viruses that maintain an avirulent phenotype and evoke a protective immune response in mice (224, 225). Similar results were also observed for the influenza viruses in other hosts, including pigs, birds, and macaques (226–229). In humans, when NS1 was completely deleted from the H1N1 influenza A virus, the virus was tested in clinical trials and was demonstrated to induce higher levels of strain-specific and cross-neutralizing antibodies in a dose-dependent manner after one dose of immunization, despite the highly attenuated replication-deficient phenotype (230). Therefore, the deletion of a JAK/STAT antagonist appears to be a promising approach for the rapid attenuation of the virulence phenotype for influenza viruses.

While the deletion of a JAK/STAT antagonist for virus attenuation appears to be promising, several issues remain unclear. First, most studies that screen for JAK/STAT antagonists from viral proteins have relied on the transient expression of a viral protein in mammalian cells. The correlation between the genotype of a virus-encoded JAK/STAT antagonist and the IFN-antagonizing phenotype of an entire virus requires further investigation. As a typical example, the comparison of the neurovirulent and attenuated variant of JEV in Stat-1-deficient mice demonstrated that the attenuated phenotype of JEV is completely lost (231), suggesting an important role for the IFN-activated JAK/STAT pathway in controlling JEV infection. However, it is notable that the NS5 protein (acting as an IFN antagonist among all flavivirus) of the attenuated JEV strain maintains its potential for antagonizing IFN similar to the neurovirulent strain. Conversely, a single Glu to Lys mutation at aa 138 in the JEV envelope protein demonstrated both IFN sensitivity and the attenuated phenotype in inoculated animals (231). Research from our lab PRRSV showed a similar result. The IFN and JAK/STAT antagonist of PRRSV was mapped to the first 4 kb of the PRRSV genome, which includes the coding region for NSP1α, NSP1β, and NSP2 (232). However, one novel PRRSV isolate, A2MC2, which maintains an IFN-inducing phenotype and does not block JAK/STAT signaling in cell culture, contains an identical sequence for the first 4 kb when compared with the PRRSV strain inhibiting IFN induction and signaling (232). Therefore, when elucidating the mechanism of the JAK/STAT IFN antagonist, data gained from artificial overexpression of putative viral JAK/STAT antagonists should be carefully reviewed, and its putative role should be further verified in virus-infected cells.

Moreover, single amino acid mutation-mediated relief of JAK/STAT antagonism and virus attenuation has been reported (219, 220). Considering the natural mutation rate of virus replication, especially for RNA viruses, the restoration of virulence from an attenuated phenotype is a significant concern, even if multiple amino acid substitutions are introduced. As a typical example, after alanine-scanning mutagenesis, PRRSV-NSP1β, a well-defined IFN-JAK/STAT antagonist encoded by PRRSV, was substituted with alanines to aa 16–20 of nsp1β in mutant PRRSV and generated a viable virus with attenuated phenotype *in vitro*. However, after infecting pigs, the recombinant virus exhibited reduced growth at early infection times but quickly regained wild-type growth properties as a result of substitutions within the mutated sequence (233), suggesting high selection pressure toward maintaining the IFN-JAK/STAT inhibitory property of the virus *in vivo*. Conversely, partial deletion of the JAK/STAT antagonist, such as a truncated influenza NS1, may be a preferred approach to single or multiple amino acid mutations. However, since a viral JAK/STAT antagonist may be indispensable for viral replication and deletion of JAK/STAT antagonist from viral genome may be lethal, introducing a non-lethal but stable deletion for a viable recombinant virus requires careful investigation and a deep understanding of viral protein function (234).

## Virus-Induced Serine Mono-Phosphorylation of STATs and Viral Pathogenesis

As discussed in Section “Viral Interference of IFN-Activated JAK/STAT,” the phosphorylation of tyrosine resides (near residue 700) in STATs is generally considered the activation of the canonical JAK/STAT pathway (31). However, mono-phosphorylation of serine residues of different STATs has been frequently reported as non-canonical TAD serine phosphorylation without tyrosine phosphorylation (31), which may imply a novel function for STATs during virus infection and pathogenesis.

Although less investigated, virus-induced serine mono-phosphorylation of STATs with different functions than tyrosine-phosphorylated STATs has been reported for both DNA and RNA viruses, such as EBV, HIV, and PRRSV (44, 235, 236). Based on our literature research, EBV was first reported for its ability to induce serine mono-phosphorylation of STAT1 (235). EBV-induced serine mono-phosphorylated STAT1 is able to bind DNA in EBV-infected cells (235). However, researchers in this study postulated that EBV uses serine mono-phosphorylation of STAT1 to restrict IFN-stimulated STAT1-DNA binding, therefore preventing IFN-activated JAK/STAT signaling. In a later study conducted on HIV-1, serine mono-phosphorylation of STAT1 and STAT3 was observed in HIV-1-infected human brain microvascular endothelial cells and correlated with HIV-1-induced inflammatory responses and neuropathogenesis (236).

Porcine reproductive and respiratory syndrome virus is known for its capability to inhibit both IFN induction and IFN-activated JAK/STAT signaling, and several PRRSV antagonists for JAK/STATs have been identified (172, 198, 237, 238). However, it is notable that PRRSV infection promotes the IFN-independent serine mono-phosphorylation of STAT1 (S727) *via* nsp12 and is linked to higher expression of proinflammatory cytokines *in vitro* (44). Moreover, mono-phosphorylation of STAT1 (S727) is correlated with viral virulence, as a vaccine strain demonstrated a minimal effect on pSTAT1-S727 (44). This observation is interesting because the PRRSV genome encodes several nsps to block both PRR signaling (TLR or RLR) and JAK/STAT signaling (238, 239). However, aberrant sustained expression of proinflammatory cytokines and chemokines is considered to contribute to the virulence of high-pathogenesis PRRSV (240). Therefore, it appears that the expression of proinflammatory cytokines and chemokines promoted by IFN-independent mono-phosphorylation of STAT1 offers an alternative explanation for the cytokine storm that occurs during PRRSV infection. However, this speculation requires further investigation.

Research on KSHV also shows that the latent protein kaposin B of KSHV promotes the mono-phosphorylation of STAT3 at S727 in the absence of the phosphorylation of Y705 (241). It appears that mono-phosphorylation of STAT3 at S727 is activated by the host kinase mitogen-activated protein kinase-activated protein kinase 2 (MK2) and leads to elevation of STAT3-dependent genes, including CCL5 (241). Moreover, kaposin B of KSHV upregulates inflammatory cytokine levels, which correlates to KS pathogenesis (242). This finding is consistent with the putative function of serine mono-phosphorylated STAT3 in HIV-1 (236).

To date, serine mono-phosphorylation induced by virus infection has only been reported in STAT1 and STAT3. The available data imply a correlation between serine mono-phosphorylation of STATs (STAT1 and STAT3) and the proinflammatory response caused by virus infection. It is still unclear whether virus-induced serine mono-phosphorylation is common among all STATs or restricted to STAT1 and STAT3, since canonical activation of STAT1 and STAT3 also induces a proinflammatory response. Moreover, it is interesting to determine whether U-ISGF3 carries mono-phosphorylated STAT1, as previous reports of U-ISGF3 only focus on tyrosine phosphorylation, without testing serine phosphorylation (36, 38). Together, the correlation of non-canonical STAT activation, serine mono-phosphorylated STATs, and unphosphorylated STATs during viral infection requires further study and may yield insights regarding viral pathogenesis, such as virally induced cytokine storms.

## Conclusion and Perspectives

The induction and signaling of type I IFNs are well-defined, and the antagonism of IFN-JAK/STAT pathway by many viruses is known. However, many questions about the type I IFN-activated JAK/STAT pathway remain unanswered. Although type I IFN subtypes appear to be functionally redundant because all type I IFNs bind to the same receptors, the differences among type I IFN subtypes are still unclear. Although activating the same pathway as type I IFNs, the function of type III IFNs requires further study. A recent report on Yellow fever virus (YFV) showed that type III IFN-mediated signaling is critical for controlling the infection of live attenuated YFV *in vivo* (243). Meanwhile, in HepG2 cells with persistent HEV infection, persistent activation of JAK/STAT signaling by type III IFNs renders the infected cells refractory to exogenous type I IFN treatment, and depletion of the receptors for type III IFNs restores IFN responsiveness (244). It would be interesting to examine the cross talk between type I and type III IFN-mediated signaling.

Although JAK/STAT antagonists have been identified for many viruses, for viruses that cause chronic infection, the role of type I IFN-activated JAK/STAT signaling in viral pathogenesis and virulence is more complicated. It appears that type I IFN-induced negative regulatory pathways are emerging as key drivers of chronic inflammation during chronic virus infection (245). During chronic HCV infection, the activation of endogenous type I IFN signaling and the elevation of hepatic ISGs contribute to HCV persistence (246). Therefore, the role of JAK/STAT in chronic viral infection should be reconsidered carefully, since the antiviral effects of type I IFNs are primarily manifested in an acute infection (247).

Virally encoded antagonists of type I IFN signaling are generally considered virulence factors that can be explored for virus attenuation. Current attenuation methods based on the mutation of type I IFN antagonists are still premature. Although reverse genetics provide a useful tool to manipulate viral genomic sequences, the restoration of a type I IFN-JAK/STAT-antagonizing phenotype in a recombinant virus during infection is a concern. As a result, a deep understanding of the structure and function of virally encoded JAK/STAT antagonists is more important than simple identification of JAK/STAT antagonists from viral genome. Furthermore, the potential link between virus-induced serine mono-phosphorylation of STATs and viral pathogenesis suggests that an interplay between viruses and the JAK/STAT pathway is more complicated than simply counteraction of each other. In the coming decade, we expect that more attention will be paid to these aspects to increase our understanding of the type I IFN-activated JAK/STAT pathway, the mechanism of viral-coded type I IFN antagonists and the role of non-canonical STAT activation in viral pathogenesis.

## Author Contributions

All authors listed have made a substantial, direct, and intellectual contribution to the work and approved it for publication.

## Conflict of Interest Statement

The authors declare that the research was conducted in the absence of any commercial or financial relationships that could be construed as a potential conflict of interest.
